# The role of volatile organic compounds for assessing characteristics and severity of non-cystic fibrosis bronchiectasis: an observational study

**DOI:** 10.3389/fmed.2024.1345165

**Published:** 2024-04-03

**Authors:** Shu-Yi Gu, Hai-Wen Lu, Jiu-Wu Bai, Jia-Wei Yang, Bei Mao, Li Yu, Jin-Fu Xu

**Affiliations:** Department of Respiratory and Critical Care Medicine, Institute of Respiratory Medicine, Shanghai Pulmonary Hospital, Tongji University School of Medicine, Shanghai, China

**Keywords:** bronchiectasis, volatile organic compounds, hypoxia, BSI, E-FACED

## Abstract

**Background:**

Hypoxic conditions and *Pseudomonas aeruginosa* (*P. aeruginosa*) infection are significant factors influencing the prognosis and treatment of patients with bronchiectasis. This study aimed to explore the potential for breath analysis to detect hypoxic conditions and *P. aeruginosa* infection in bronchiectasis patients by analyzing of volatile organic compounds (VOCs) in exhaled breath condensate (EBC).

**Methods:**

EBC samples were collected from stable bronchiectasis patients and analyzed using solid phase microextraction-gas chromatography-mass spectrometry (SPME-GCMS). The association of VOCs with bronchiectasis patients' phenotypes including hypoxic conditions and *P. aeruginosa* isolation was analyzed, which may relate to the severity of bronchiectasis disease.

**Results:**

Levels of 10-heptadecenoic acid, heptadecanoic acid, longifolene, and decanol in the hypoxia group were higher compared to the normoxia group. Additionally, the levels of 13-octadecenoic acid, octadecenoic acid, phenol, pentadecanoic acid, and myristic acid were increased in *P. aeruginosa* (+) group compared to the *P. aeruginosa* (–) group. Subgroup analysis based on the bronchiectasis severity index (BSI)reveled that the levels of 10-heptadecenoic acid, heptadecanoic acid, decanol, 13-octadecenoic acid, myristic acid, and pentadecanoic acid were higher in the severe group compared to the moderate group. Multivariate linear regression showed that 10-heptadecenoic acid and age were independent prognostic factors for bronchiectasis patients with hypoxia. Furthermore, octadecenoic acid, phenol and gender were identified as independent prognostic factors for bronchiectasis patients with *P. aeruginosa* isolation.

**Conclusion:**

The study provides evidence that specific VOCs in EBC are correlated with the severity of bronchiectasis, and 10-heptadecenoic acid is shown to be a predictive marker for hypoxia condition in bronchiectasis patients.

## 1 Introduction

Bronchiectasis is a chronic respiratory disease that occurs due to respiratory inflammation and structural damage to the bronchus and its surrounding lung tissue, which destroys the muscle and elastic tissue of the bronchial wall, resulting in bronchial deformation and lasting expansion. The disease is characterized by recurrent cough, sputum production, and respiratory tract infections (1–3). Bacterial infections trigger an inflammatory response, which further exacerbates airway inflammation and airway structural damage, leading to the well-described “vicious cycle” of bronchiectasis (4, 5).

The definition of EBC by the previous ERS/American Thoracic Society (ATS) TF is used (6). Briefly, EBC is obtained by cooling exhaled breath through contact with a cold surface or condenser. Samples are collected as fluid or frozen material and analyzed immediately or later for volatile and non-volatile macromolecules. VOCs are carbon-based chemicals, especially in a volatile state at ambient temperature, originating from physiological and pathophysiological metabolic processes in the human body. Their origin may be local, systemic, and exogenous (7). Analysis of exhaled breath has been used to detect and monitor diverse pulmonary and systemic diseases such as oxidant-induced airway injury (8), aspirin-induced asthma (9), lung cancer (10, 11), chronic obstructive pulmonary disease (12), tuberculosis (13), lung transplant rejection (14), breast cancer (15), heart transplant rejection (16), diabetes mellitus (17), and unstable angina (18). Mansoor et al. used non-invasive exhaled breath condensate (EBC) analysis to identify potential novel biomarkers that correlated with IPAH pulmonary hemodynamic variables that may be important in screening for less severe forms IPAH (19). A previous study characterized VOCs in a broad range of human samples and identified more than 1,840 different compounds (20). Yamada et al. used to identify five specific VOCs in the exhaled breath of ILD patients, suggesting that measurement of VOCs promise for discriminating ILD patients from healthy controls (21). Horck et al. proposed a distinct breath VOC pattern that aids in diagnosing children with cystic fibrosis (22). Kamal et al. reported virus-induced VOCs in EBC during pulmonary infection (23), indicating that microorganism metabolism. Acid gastroesophageal reflux is a common problem in non-cystic fibrosis bronchiectasis, Annemarie et al. detected pulmonary microaspiration is to measure pepsin in exhaled breath condensate measure pepsin concentrations and pH in EBC and to determine the relationship to gastroesophageal reflux in bronchiectasis (24). Karakoc et al. evaluate the MMP-9 and its natural tissue inhibitors of metalloproteinases (TIMP-1) levels utilizing the exhaled breath condensate (EBC) method and their relationship with radiological findings and pulmonary functions in children with bronchiectasis (25). Savelev et al. found 2-Nonanone is a compound in the exhaled breath as it may improve diagnostic of Ps. aeruginosa infection when combined with other reported volatile markers (26). Moreover, Bikov et al. put forward an opinion that exercise-caused metabolic changes can be followed by monitoring exhaled volatiles and their study shows that physical exercise causes a change in exhaled breath volatile compound pattern, which presumably reflects an increase in systemic and local metabolism in the airways (27). They also indicated that the dilution of airway lining fluid (ALF) acids and bases by alveolar water may influence condensate pH (28). However, limited research has been conducted to elucidate the significance of VOCs in human breath samples for evaluating and monitoring disease progression.

The present study aimed to analyze the relationship between VOCs in the EBC of bronchiectasis patients and their hypoxic conditions. Additionally, the correlation between specific VOCs and the severity of bronchiectasis was also explored. All EBC samples were analyzed by SPME-GC/MS technology.

## 2 Materials and methods

### 2.1 Patients

**Figure 1:**
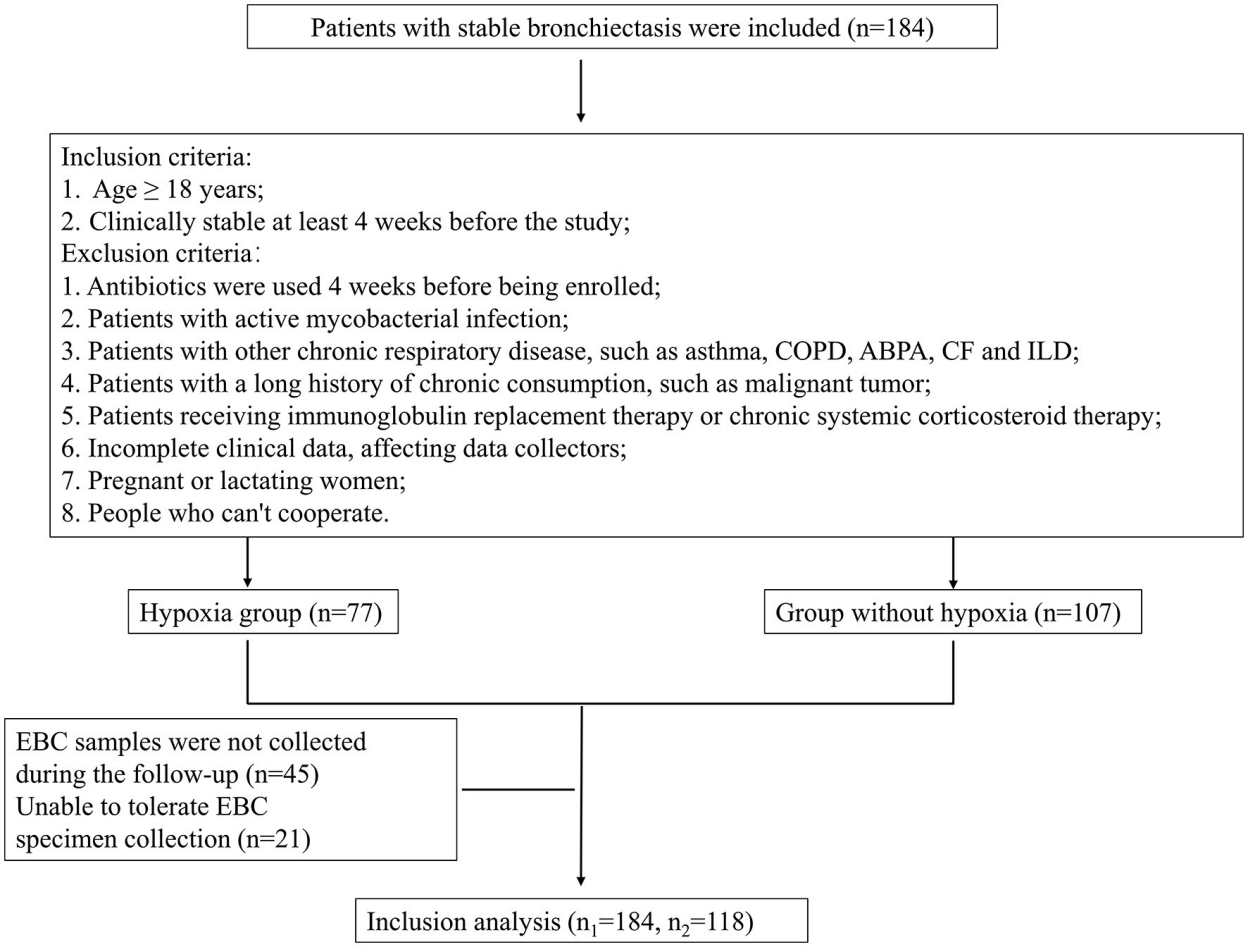
Detailed procedures of the study and EBC sample entry criteria. COPD, Chronic obstructive pulmonary disease; ABPA, Allergic bronchopulmonary aspergillosis; CF, Cystic fibrosis; ILD, Interstitial lung disease; EBC, Exhaled breath condensate.

A total of 184 stable bronchiectasis patients were recruited from the Shanghai Pulmonary Hospital (Shanghai, China) from September 2021 to October 2022. Out of these, 118 patients collected EBC samples. Detailed procedures of the study are shown in Figure 1. The present study was approved by the Ethics Committee of Shanghai Pulmonary Hospital (Approval No. K21-316). Detailed inclusion and exclusion criteria can be found in Figure 1. Patients were categorized into two groups according to whether they had hypoxia or whether *P. aeruginosa* was isolation from sputum.

### 2.2 Clinical and functional assessment

All patients underwent chest high-resolution computed tomography (HRCT), sputum culture test, arterial blood gas test, and pulmonary function tests. Bronchiectasis severity index (BSI) and exacerbation frequency, forced expiratory volume in 1 second, age, colonization, extension and dyspnea (E-FACED) score were used to assess the severity of bronchiectasis. Patients were grouped based on blood oxygen saturation (SPO_2_) and microbial culture results. Patients with SPO_2_ < 90% were assigned to the hypoxia subgroup, and those with SPO_2_ ≥ 90% were allocated to the normoxia subgroup. The *P. aeruginosa* (+) subgroup was defined as *P. aeruginosa* isolation from patients' sputum culture, otherwise, patients were assigned to *P. aeruginosa* (–) subgroup.

### 2.3 EBC collection

In this study, exhaled gas samples were collected using the RTubeVOC™ End Tidal Air Collector (Respiratory Research, Inc., Austin, TX, USA), the entire collection system especially for VOCs examination. The entire collection system was cooled in −20°C for at least 24 h before sample taken. Subjects were instructed to eat a light diet at the night and breakfast was not allowed before sampling, while smoking and brushing were also forbidden. It is advised that subjects refrain from exercise for at least 1 h preceding EBC collection (29). The subjects sat quietly in the same sampling room for ≥5 min. Microbial activity in the oropharyngeal tract significantly contributes to the concentration of nitrogen oxides in EBC (30), so the subjects should rinse mouth with clean water for three times before sample taken. The subjects were instructed to breathe quietly (tidal breathing) for 5 min and the use of a nose clip is advised. During the period periodic swallowing is advised for limitation of salivary contamination. Then, their exhaled gas samples were collected using the RTubeVOC™ End Tidal Air Collector which is end tidal air collector for Volatile Organic Compounds and disposable single-breath collector selectively captures the last 65 ml of exhalate. This unique feature allows for easy integration of the RTubeVOC into deep-lung VOC studies. EBC volume ranged 1.5–2 mL, the subject would be asked repeat collecting if EBC volume was < 1.5 ml. All the Rtube exhaled breath condensate collector were disposable design ensures a clean device ready for use at any time with no risk of infectious disease transmission between patients. Samples were stored by sealable collection tube immediately after collection (in order to avoid any errors by evaporation of volatile components), and storage at −80°C until analyzed. All the samples were sent to further analysis within 2 weeks.

### 2.4 Solid phase microextraction-gas chromatography-mass spectrometry

The EBC sample was placed in a Clear Crimp Top Vial (20 mL) (Thermo Scientific™, USA) by using Universal Needle Interface (Respiratory Research, Inc., Austin, TX, USA). The vial was sealed using a crimping machine (CRIMPER 20 mm, Japan) and placed in a thermostat at 40°C. The pre-activated solid-phase microextraction (SPME) device was inserted, push out the extraction head, recover the extraction head after headspace extraction for 25 min, and wait for sample introduction and analysis.

A manual SPME holder and a commercial SPME fiber: 50/30-μm poly(acrylate) (PA), were purchased from Supelco company (USA). The SPME fiber was conditioned to recommended degrees before it was used for the first time. The fiber, first desorbed at 230°C for 5 min in the GC injector, was exposed in the headspace of the vial for 25 min to adsorb the volatile organic compounds from the sample; then it was removed from the vial and introduced into the GC injector for 3 min where the thermal desorption of the analytes was carried out. Instruments used for detection included Thermo Trace ISQ gas chromatograph-mass spectrometer (Thermo Scientific™, USA), which was equipped with TriPlus autosampler and ACEM9300 thermal desorption instrument (Thermo Scientific™, USA), as well as an HP-INNOWax quartz capillary column (30 m × 0.32 mm, 0.50 μm) as a chromatographic column. Chromatographic conditions were as follows: Column temperature: the initial temperature was 60°C for 3 min, which was increased to 240°C at 15°C min-1, 240°C was maintained for 10 min; inlet temperature was 230°C; Carrier gas: He (>99.999%), with a flow rate of 1 mL/min. Splitless mode was used. The mass spectrometry conditions were as follows: Ionization source: EI source, ionization energy of 70 eV, and ion source temperature of 250°C; Transmission line temperature: 230°C; Scan range: 41–400 amu; The compounds were identified using NIST14 Mass Spectral Search Program (National Institute of Standards and Technology, Washington, DC, USA).

### 2.5 Plasma inflammatory factor

At the time of clinical assessment, each subject underwent a blood sample collection. Venous blood was drawn by venipuncture from all subjects in the morning after an overnight fast, 1 ml of blood per patient. Plasma samples were collected in EDTA vacutainers, 50 μl for each sample, which were immediately centrifuged for 15 min at ~2,000 × g at room temperature. Then, plasma inflammatory factor indexes (IL-1β, IL-6, IL-2R, TNF-α) were analyzed with flow cytometry (BD FACSCantoTM II, USA) through a panel of four cytokines kit, namely human IL-1β, IL-6, IL-2R, TNF-α (Genebio P010104007, China) and converted in picograms per milliliter.

### 2.6 Statistical analysis

Gas chromatography and mass spectrometry combining computer retrieval with NIST 14 was used for identifying the VOCs. Half-quantitative analysis was achieved through comparison of peak absolute intensities and peak area normalization method was used for calculating their relative content of compositions. All VOC-related data were exported to SIMCA 14.1 software (MKS Umetrics, Umeå, Sweden) for principal component analysis (PCA) and orthogonal partial least squares discriminant analysis (OPLS-DA). The variable importance for projection (VIP) in the OPLS-DA was calculated and selected characteristic ions that met the criterion of VIPs >1.0 were regarded as potential targets for further analysis. The Chi-square test was used for the analysis of categorical data.

Continuous variables were expressed as mean ± standard deviation (SD) or median (interquartile range), and categorical variables were presented as counts or percentages (%). The Student's *t*-test, Wilcoxon test, or the Mann-Whitney U test was employed for analyzing normally or non-normally distributed continuous data of two or three independent groups. The Spearman's correlation was used to analyze the relationships between VOCs and BSI and E-FACED scores in all patients. The univariate and multivariate linear analyses of bronchiectasis patients with or without hypoxia were conducted. The log transformation was also performed on the values of the above-mentioned parameters to carry out linear regression analysis again. Data analysis was conducted using SPSS 26.0 (IBM Corp., Armonk, NY, USA), and GraphPad Prism 9.0 (GraphPad Software Inc., San Diego, CA, USA) software.

## 3 Results

**Figure 2:**
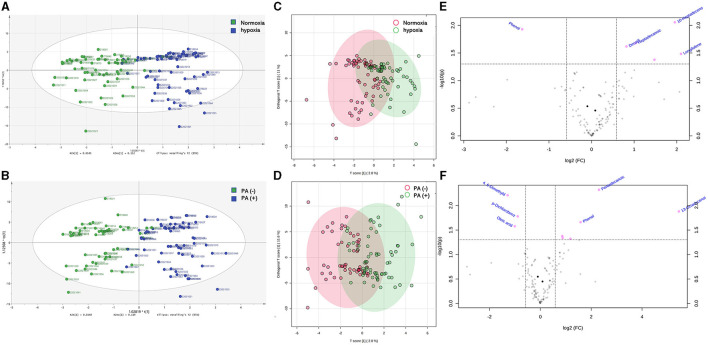
Differences of inflammatory factors, arterial oxygen partial pressure and oxygen saturation in peripheral blood between *P. aeruginosa* (–) group and *P. aeruginosa* (+) group. The PaO_2_ in P. aeruginosa (+) group (*n* = 54) were significantly lower than those in P. aeruginosa (–) group (*n* = 64), while the levels of plasma inflammatory factors showed no statistically significant in two groups. IL-1β, Interleukin-1β; IL-6, Interleukin-6; SIL-2R, soluble interleukin 2 receptor; TNF-α, Tumor necrosis factor-α. Boxplot whiskers show median ± Tukey distribution, *P. aeruginosa* (–) group: *n* = 54, *P. aeruginosa* (+) group: *n* = 64. Student's *T*-test was performed for **(A–F)**.

The rate of *P. aeruginosa* isolation increased in bronchiectasis patients with hypoxemia. Based on the results of *P. aeruginosa* isolation, 184 patients with bronchiectasis were divided into two groups, 79 patients in *P. aeruginosa* (+) group and 105 patients in *P. aeruginosa* (–) group. The differences in inflammatory factors, arterial oxygen partial pressure (PaO_2_) and arterial oxygen saturation (SaO_2_) between the two groups were compared. As shown in Figure 2, we found that the levels of inflammatory factors in the peripheral blood of patients with bronchiectasis were higher in *P. aeruginosa* (+) group than in *P. aeruginosa* (–) group, such as interleukin-1β (IL-1β), interleukin-6 (IL-6), soluble interleukin 2 receptor (SIL-2R) and tumor necrosis factor-α (TNF-α). However, the differences in these levels were not statistically significant (*P* > 0.05). The PaO_2_ in *P. aeruginosa* (+) group were significantly lower than those in *P. aeruginosa* (–) group (*P* < 0.05). Because SpO_2_ in patients can be affected by a variety of factors, such as poor fingertip tip circulation and nail fungal infection, this study did not suggest that there was a difference in SpO_2_ between subjects.

### 3.1 Baseline characteristics

A total of 118 patients with bronchiectasis were enrolled in the EBC sample collection, and their clinical characteristics were summarized in Table 1. The patients were divided into two groups according to whether they had hypoxia or isolation of *P. aeruginosa*. Among them, 56 patients had normoxia, while 62 patients had hypoxia. Additionally, 54 patients were in *P. aeruginosa* (–) group, and 64 patients were in *P. aeruginos*a (+) group. Significant differences were observed in age and gender between these two groups while there was no significant different. The hypoxia group exhibited a significantly lower FEV1% compared to the normoxia group. However, no significant difference was found in FEV1% between the *P. aeruginosa* (+) group and the *P. aeruginosa* (–) group. Importantly, both BSI and E-FACED scores were higher in the hypoxia and *P. aeruginosa* (+) groups compared to the normoxia and PA (–) groups, indicating that patients in the hypoxia group or *P. aeruginosa* (+) group experienced from a more severe disease stage.

**Table 1:** Characteristic of patients with bronchiectasis.

	**Normoxia (*n* = 56)**	**Hypoxia (*n* = 62)**	***P* value**	***PA* (–) (*n* = 54)**	***PA* (+) (*n* = 64)**	***P* value**
**Age (years old)**	51.23 (48.01,54.45)	58.05 (55.77,60.33)	0.002	58.22 ± 11.66	58.94 ± 9.67	0.002
**Gender**			0.002			0.003
Men	16 (28.66)	28 (45.2)		28 (51.9	16 (25.0)	
Female	40 (71.4)	34 (54.8)		26 (48.1)	48 (75.0)	
**Smoking history**			0.466			0.017
Yes	8 (14.3)	12 (19.4)		14 (25.9)	6 (9.4)	
No	48 (85.7)	50 (80.6)		40 (74.1)	58 (90.6)	
**BMI**	21.16 (20.47,21.86)	22.23 (21.18,23.28)	0.307	21.06 ± 23.49	22.28 ± 3.4	0.062
**FEV1%**	66.26 ± 21.49	50.81 ± 18.76	< 0.001	61.10 ± 20.55	55.65 ± 22.05	0.171
**BSI**			0.035			0.000
1	3 (5.4)	2 (3.2)		5 (9.3)	0 (0)	
2	24 (42.9)	16 (25.8)		29 (53.7)	11 (17.2)	
3	29 (51.8)	44 (71)		20 (37.0)	53 (82.8)	
**E-FACED**			0.003			0.000
1	27 (48.2)	16 (25.8)		35 (64.8)	8 (12.5)	
2	26 (46.4)	34 (54.8)		18 (33.3)	42 (65.6)	
3	3 (5.4)	12 (19.4)		1 (1.9)	14 (21.9)	

BMI, Body mass index; FEV_1_%, Forced expiratory volume in the first second; BSI, Bronchiectasis severity index; E-FACED, Forced expiratory volume in 1 (FEV_1_), age, chronic colonization by *P. aeruginosa*, radiological extension and dyspnea plus exacerbations.

### 3.2 The result of VOCS in EBC of bronchiectasis patients by SPME-GC/MS

A total of 164 VOCs were detected in the EBC of all patients by using SPME-GC/MS. The acid compounds detected included myristic acid, pentadecanoic acid, hexadecanoic acid, heptadecylic acid, hexadecenoic acid, and others. The aldehyde compounds detected were as follows: benzaldehyde, dodecanal, nonanal, decanal, and others. Alcohols included 2, 2, 4-trimethyl-, 1-isoburtyrate-1, 3-pentanediol, dihydrocitronellol, 6-methyl-1-octanol, nonanol, and others.

**Figure 3:**
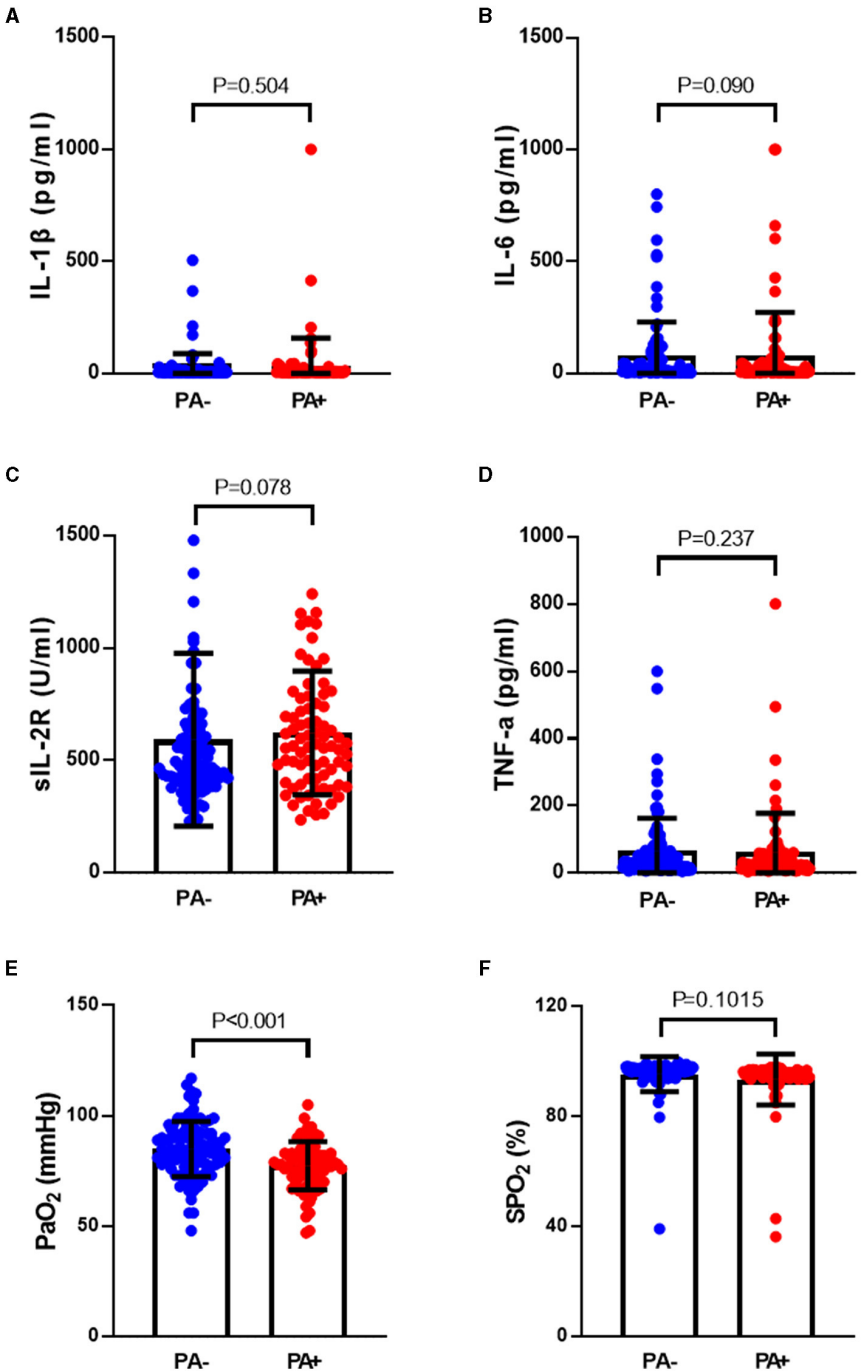
**(A)** OPLS-DA analysis results identified differences between bronchiectasis patients in hypoxia group and normoxia group, the green points showed normoxia group and the blue points showed hypoxia gruop; **(B)** OPLS-DA analysis results identified differences between bronchiectasis patients in *P. aeruginosa* (+) group and *P. aeruginosa* (–) group, the green points showed *P. aeruginosa* (–) group and the blue points showed *P. aeruginosa* (+) group; **(C)** the overall distribution and dispersion degree between hypoxia and normoxia groups, the red points showed hypoxia group and the green points showed normoxia group; **(D)** the overall distribution and dispersion degree between *P. aeruginosa* (+) group and *P. aeruginosa* (–) group, the red points showed *P. aeruginosa* (–) group and the green points showed *P. aeruginosa* (+) group; **(E)** volcano plots of different VOCs in bronchiectasis patients with hypoxia and normoxia, and the red points showed variable importance for projection (VIP) compounds; **(F)** volcano plots of different VOCs in bronchiectasis patients in *P. aeruginosa* (+) group and *P. aeruginosa* (–) group, and the red points showed variable importance for projection (VIP) compounds. Normoxia group: *n* = 56, Hypoxia group: *n* = 62. *P. aeruginosa* (–) group: *n* = 54, *P. aeruginosa* (+) group: *n* = 64. Orthogonal partial least squares discriminant analysis (OPLS-DA) performed for **(A–D)**, Principal component analysis (PCA) for **(E, F)**.

Then, the OPLS-DA analysis was conducted to evaluate the overall distribution and dispersion degree among the samples. The results revealed significant differences in VOCs in the breath samples between hypoxia and normoxia groups, as well as between the *P. aeruginosa* (+) and *P. aeruginosa* (–) groups (Figures 3A–D). The differences in VOCs between the groups were visually represented using the enhanced efficacy of the model (Figures 3E, F).

**Figure 4:**
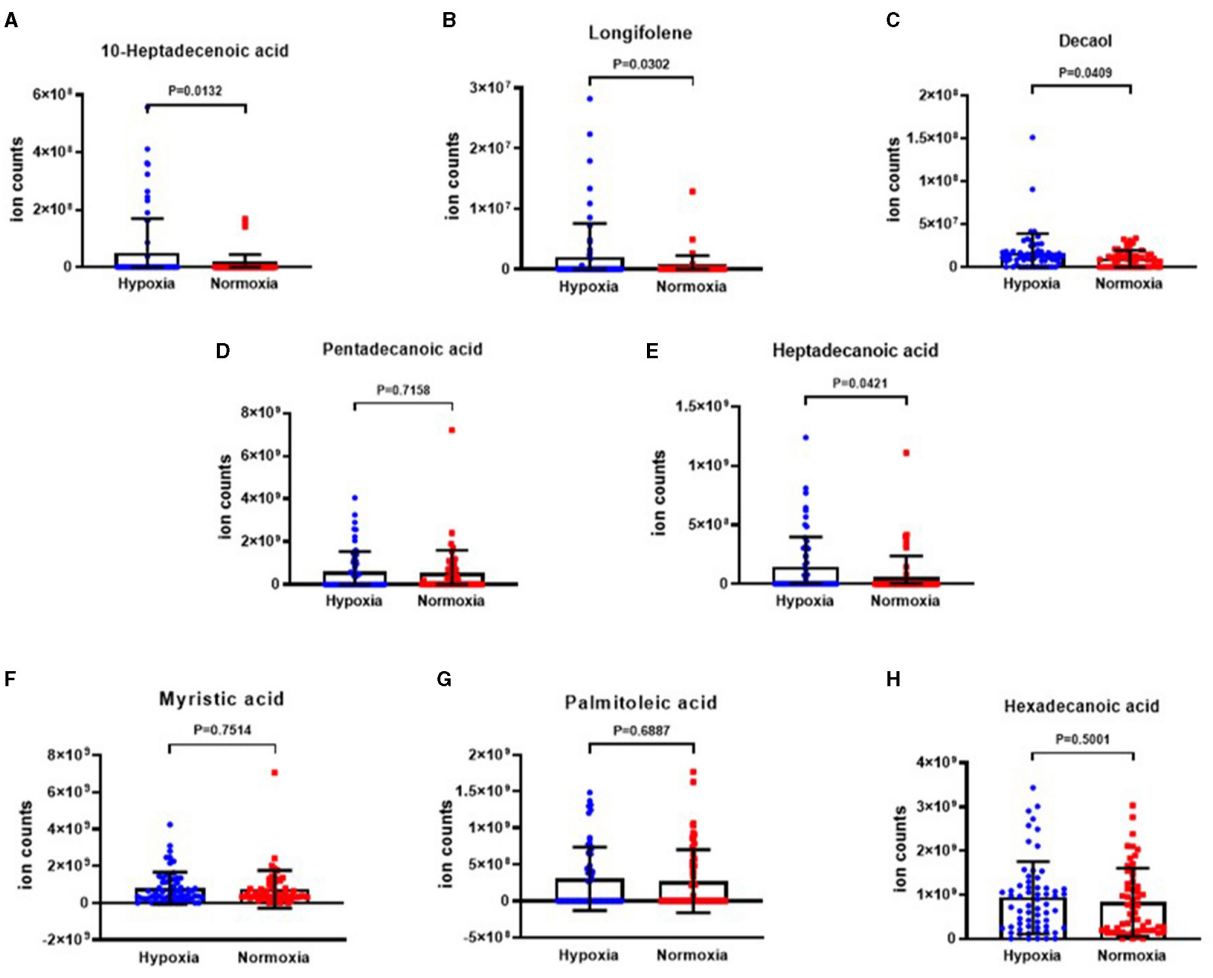
The ion counts of differential VOCs in bronchiectasis patients in hypoxia group and normoxia group. Boxplot whiskers show median ± Tukey distribution, Normoxia group: *n* = 56, Hypoxia group: *n* = 62. Student's *T-*test was performed for **(A–H)**.

**Figure 5:**
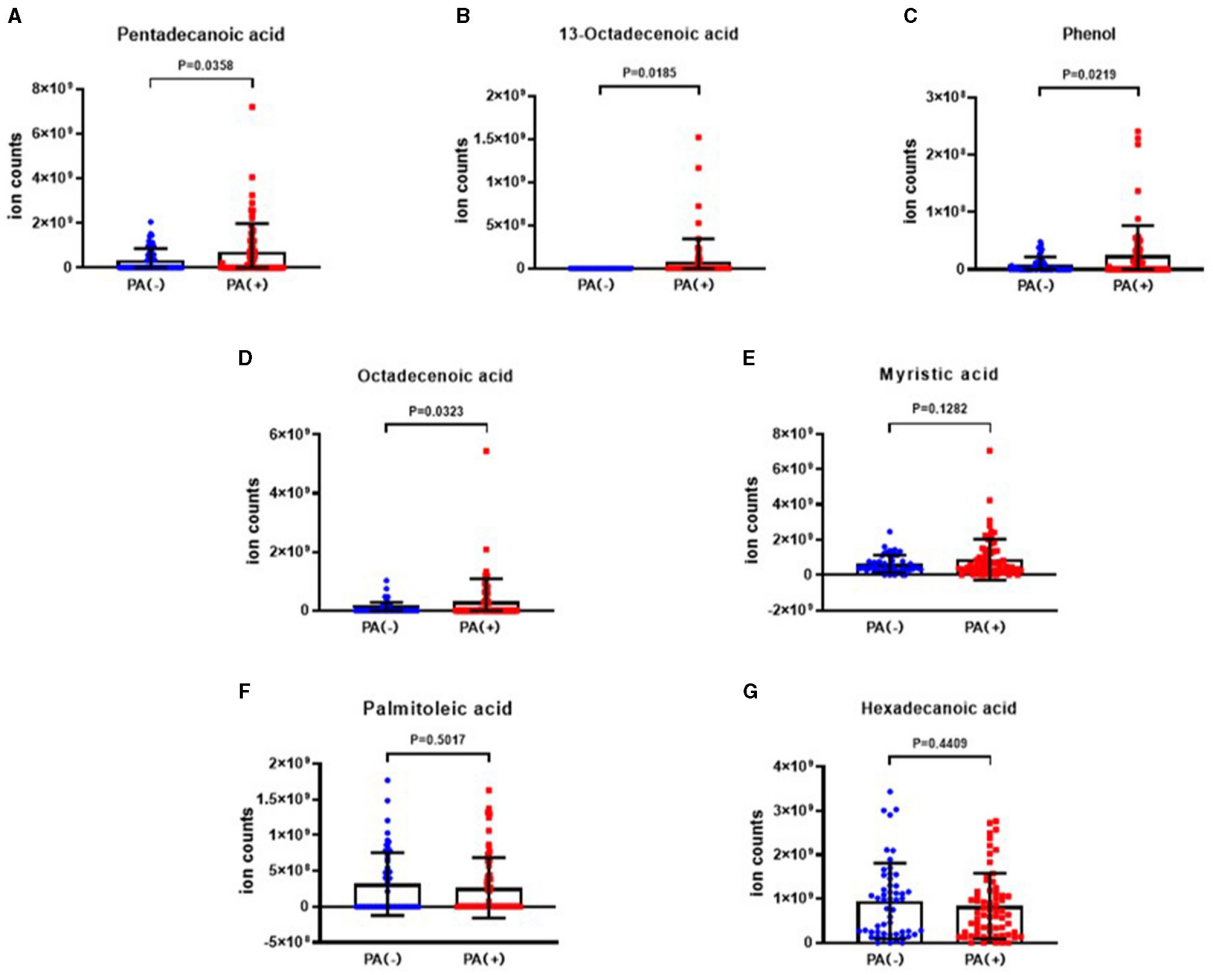
The ion counts of differential VOCs in bronchiectasis patients in *P. aeruginosa* (+) group and *P. aeruginosa* (–) group. Boxplot whiskers show median ± Tukey distribution, *P. aeruginosa* (–) group: *n* = 54, *P. aeruginosa* (+) group: *n* = 64. Student's *T*-test was performed for **(A–G)**.

The analysis revealed that the levels of 10-heptadecenoic acid, heptadecanoic acid, longifolene, and decanol were higher in the hypoxia group compared to the normoxia group, which aligned with the detection results. However, no significant differences were observed in the levels of pentadecanoic acid, myristic acid, palmitoleic acid, and hexadecanoic acid (Figures 4A–H). When comparing the different VOCs between the *P. aeruginosa* (+) and *P. aeruginosa* (–) groups, it was found that the levels of 13-octadecenoic acid, octadecenoic acid, pentadecanoic acid, phenol, and myristic acid were higher in the *P. aeruginosa* (+) group. Conversely, no significant differences were observed in the levels of palmitoleic acid and hexadecanoic acid between the groups (Figures 5A–G).

### 3.3 Differential VOCs in BSI risk stratification

**Figure 6:**
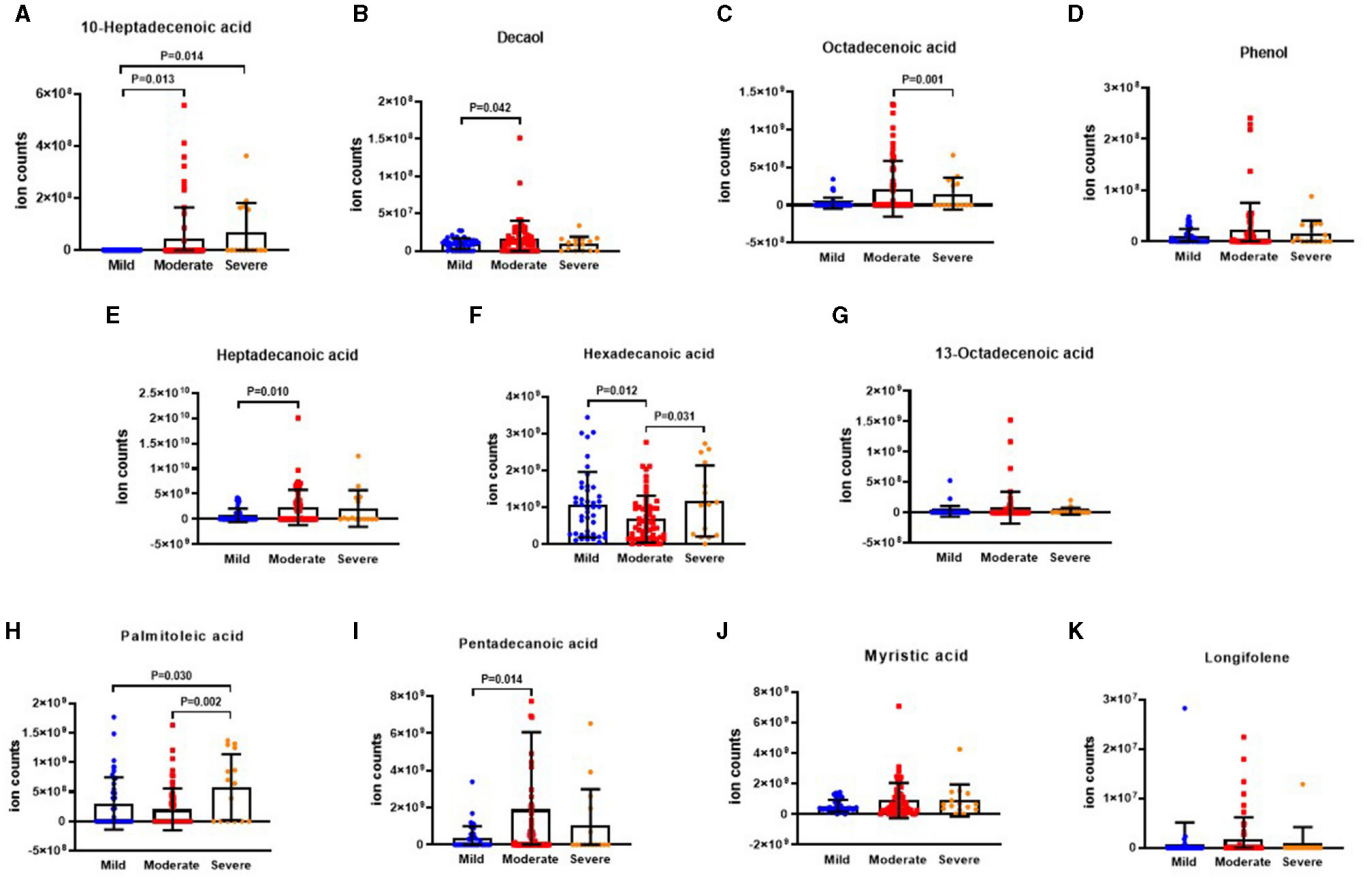
The ion counts of differential VOCs in BSI risk stratification. BSI: Bronchiectasis severity index. Boxplot whiskers show median ± Tukey distribution, Mild group: *n* = 5, Moderate group: *n* = 40, Severe group: *n* = 73. One way ANOVA with Tukey *post-hoc* test was performed for **(A–K)**.

The results depicting differential VOCs in BSI risk stratification are presented in Figures 6A–K. It was observed that the levels of 10-heptadecenoic acid, heptadecanoic acid, decanol, 13-octadecenoic acid, myristic acid, and pentadecanoic acid were higher in the severe group compared to the moderate group. Additionally, the level of octadecenoic acid was higher in the moderate group than that in the mild group. However, the level of longifolene was the lowest in the mild group when compared to the moderate and severe groups. The differences in phenol, hexadecanoic acid, and palmitoleic acid were not found to be significant among the three groups.

### 3.4 Differential VOCs in E-FACED risk stratification

**Figure 7:**
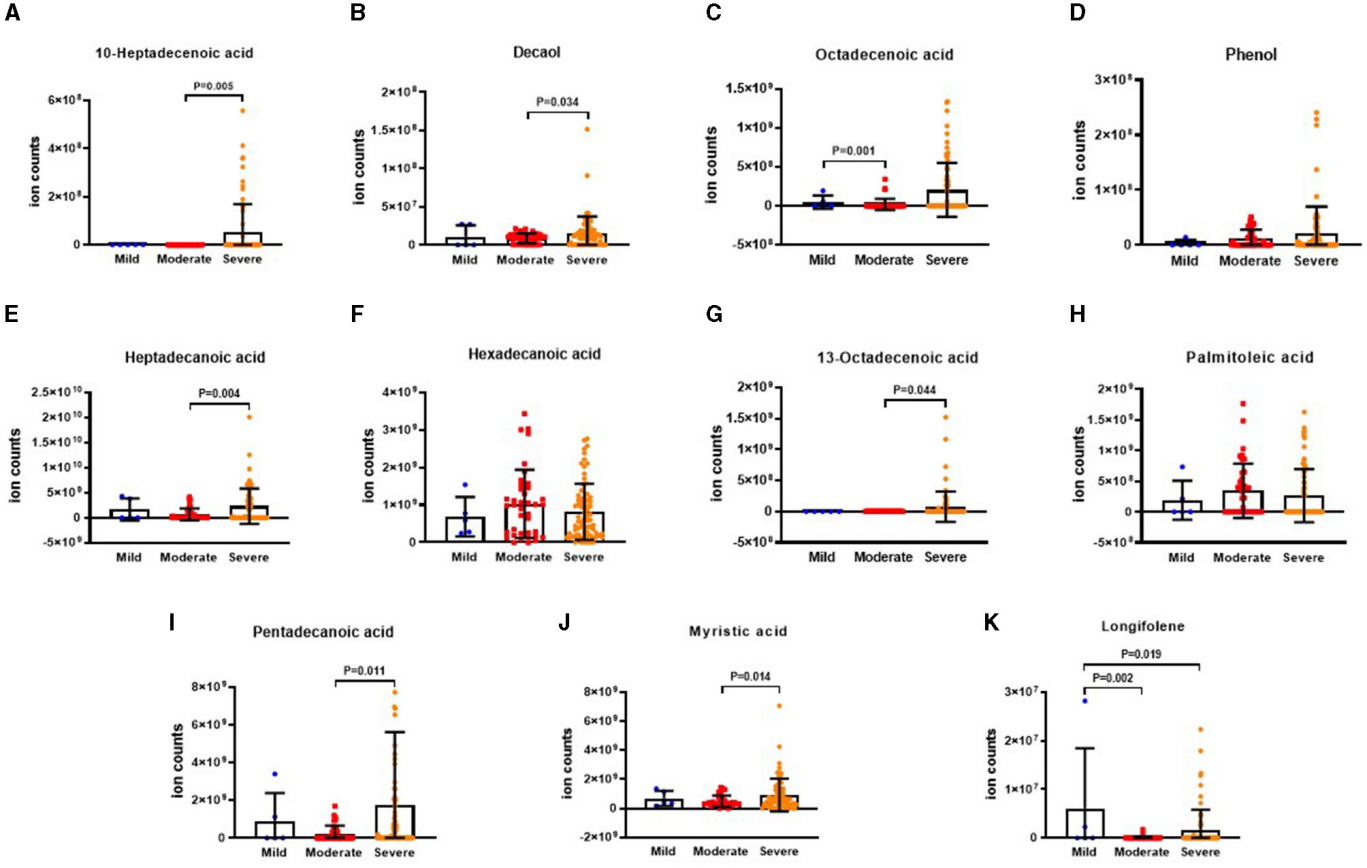
The ion counts of differences VOCs in E-FACED risk stratification. E-FACED: exacerbation frequency, forced expiratory volume in 1 second, age, colonization, extension and dyspnea score. Boxplot whiskers show median ± Tukey distribution, Mild group: *n* = 43, Moderate group: *n* = 60, Severe group: *n* = 15. One way ANOVA with Tukey *post-hoc* test was performed for **(A–K)**.

The results illustrating the differential VOCs in E-FACED risk stratification are presented in Figures 7A–K. It was observed that the level of 10-heptadecenoic acid was significantly lower in the mild group compared to the moderate and severe groups. Furthermore, the levels of decanol, heptadecanoic acid, myristic acid, and pentadecanoic acid were lower in the mild group compared to the moderate group. In addition, the octadecenoic acid level was higher in the moderate group than that in the severe group. The levels of hexadecanoic acid and palmitoleic acid were lower in the moderate group than those in the mild and severe groups. However, there were no significant differences in the levels of phenol, 13-octadecenoic acid, myristic acid, and longifolene among the three groups.

### 3.5 Relationship between VOCs and BSI and E-FACED scores

**Figure 8:**
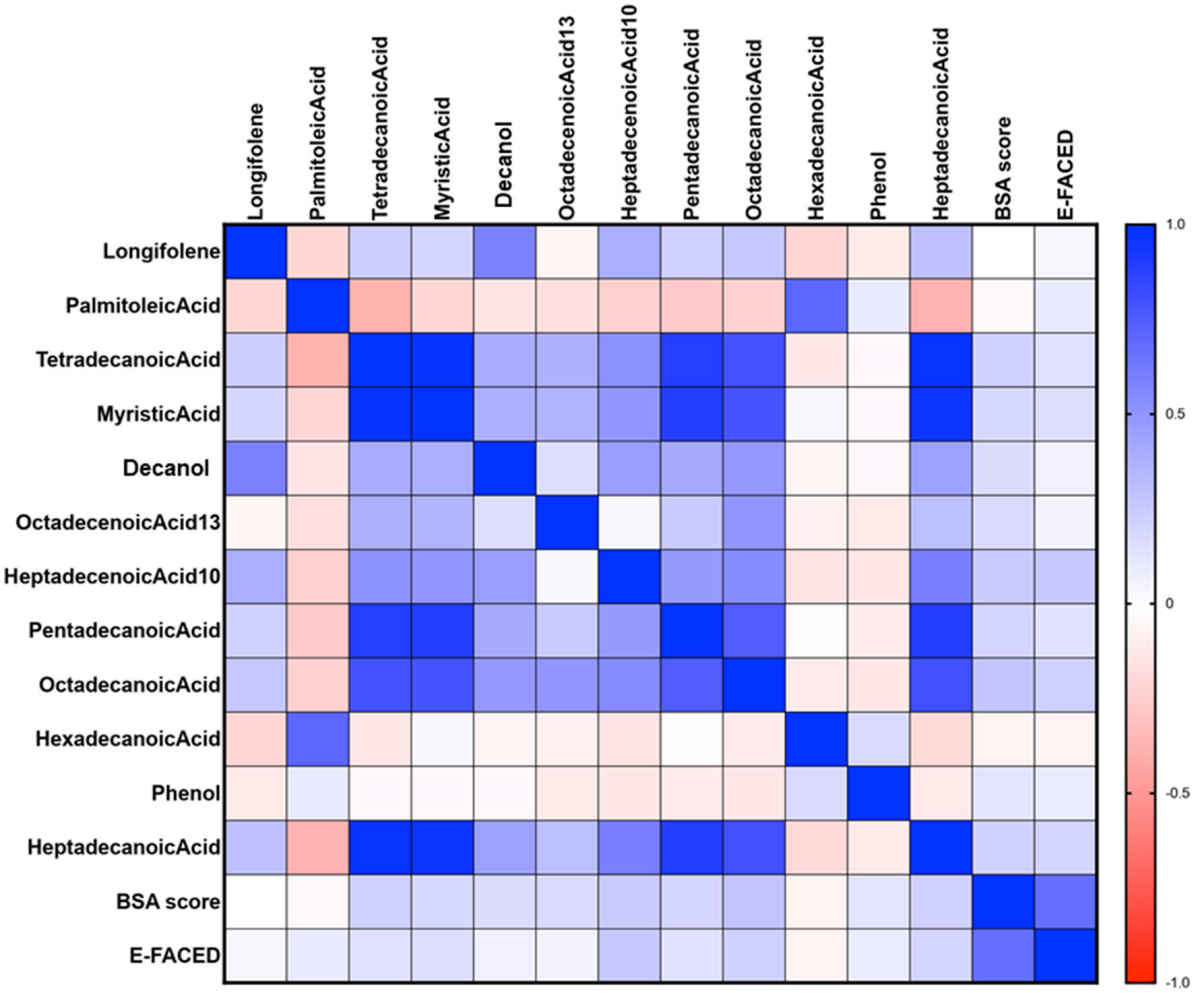
The relationship between VOCs and BSI and E-FACED scores in all patients. The Spearman's correlation analysis revealed a weak positive correlation between the BSI score and the levels of myristic acid, 13-octadecenoic acid, 10-heptadecenoic acid, pentadecanoic acid, octadecenoic acid, and heptadecanoic acid (all *P* < 0.05). Additionally, the E-FACED score exhibited a slight positive correlation with the levels of 10-heptadecenoic acid, heptadecanoic acid, and octadecenoic acid (all *P* < 0.05). Spearman's correlation was performed.

The relationships between VOCs and BSI and E-FACED scores in all patients are illustrated in Figure 8. The Spearman's correlation analysis revealed a weak positive correlation between the BSI score and the levels of myristic acid, 13-octadecenoic acid, 10-heptadecenoic acid, pentadecanoic acid, octadecenoic acid, and heptadecanoic acid (all *P* < 0.05). BSI risk stratification showed a negative correlation with the levels of longifolene, palmitoleic acid and hexadecanoic acid, but the differences were not statistically significant. Additionally, the E-FACED score exhibited a slight positive correlation with the levels of 10-heptadecenoic acid, heptadecanoic acid, and octadecenoic acid (all *P* < 0.05), while no significant correlation was observed between other VOCs and E-FACED score.

### 3.6 10-heptadecenoic acid was found as an independent prognostic factor for bronchiectasis patients with hypoxia

**Figure 9:**
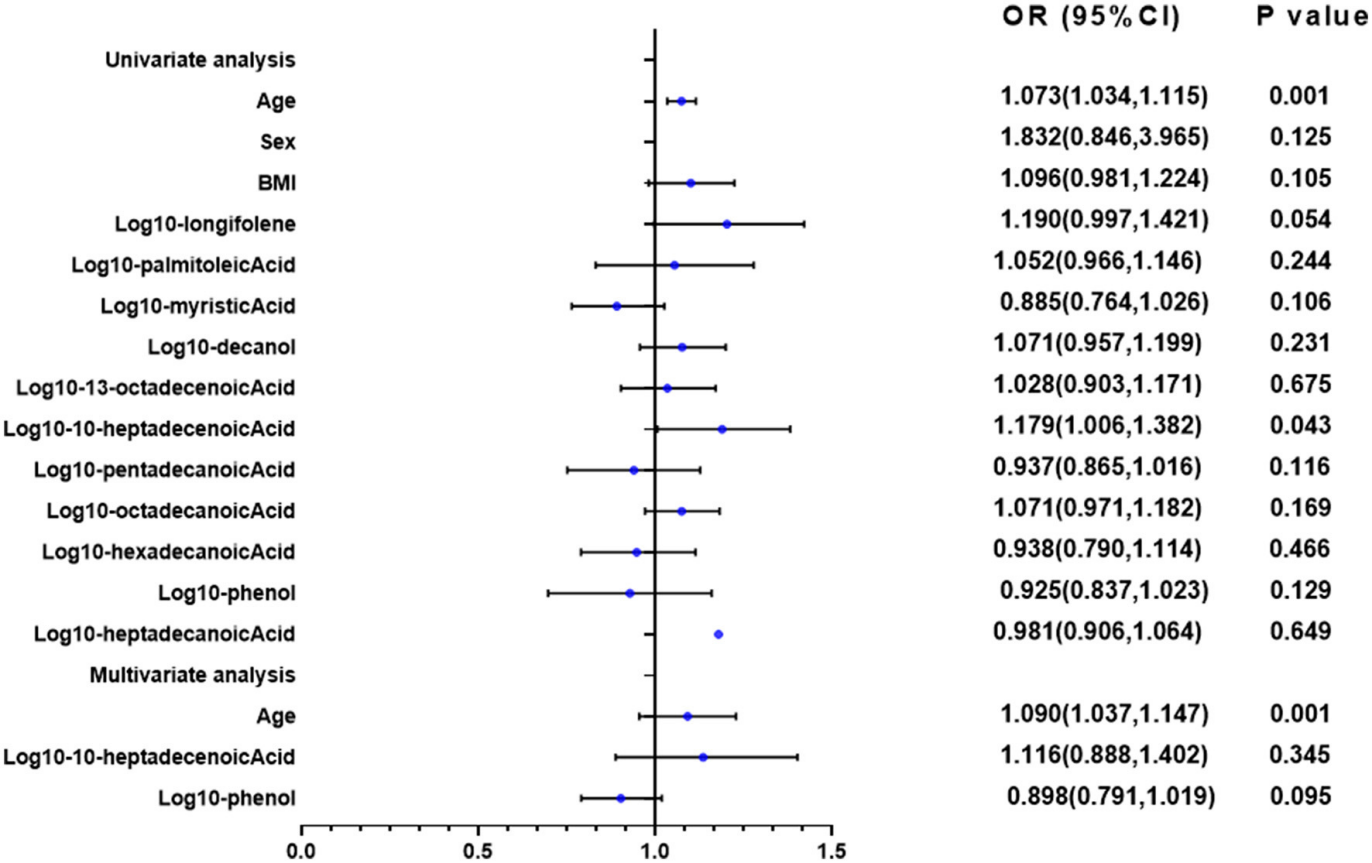
Univariate and multivariate linear regression analyses of PaO_2._ In the univariate linear analysis, age, and 10-heptadecenoic acid were found to significantly affect the hypoxic conditions of all patients (all *P* < 0.05). Univariate and multivariate linear analyses were performed.

The univariate and multivariate linear analyses were used to assess the bronchiectasis patients with or without hypoxia (Figure 9). The log transformation was performed on the aforementioned parameters for further linear regression analysis. In the univariate linear analysis, age and10-heptadecenoic acid were found to significantly affect the hypoxic conditions of all patients (all *P* < 0.05). The results of the multivariable linear analysis demonstrated that age was significantly associated with the hypoxic conditions. It was suggested that 10-heptadecenoic acid and age could serve as independent prognostic factors for bronchiectasis patients with hypoxia.

## 4 Discussion

The present study investigated the relationship between VOCs in EBC of bronchiectasis patients and disease severity. It also identified specific VOCs that predicted the risk of hypoxia and P. aeruginosa isolation in bronchiectasis patients. The BSI and E-FACED scores were higher in the hypoxia and *P. aeruginosa* (+) groups compared to the normoxia and *P. aeruginosa* (–) groups, indicating that patients with hypoxia and *P. aeruginosa* isolation experienced more severe disease. Notably, the VOC profile differed between the P. aeruginosa (+) and P. aeruginosa (–) groups, as well as between the hypoxia and normoxia groups in bronchiectasis patients. Risk stratification of bronchiectasis patients using BSI and E-FACED scores revealed that the level of 10-heptadecenoic acid was the lowest in the mild group, while the level of octadecenoic acid was higher in the moderate group compared to the severe group. Correlation analysis indicated that most of the different VOCs showed positive correlation with BSI and E-FACED scores. Furthermore, we found that 10-heptadecenoic acid and age were independent prognostic factors for bronchiectasis patients with hypoxia, while octadecenoic acid, phenol and gender were independent prognostic factors for bronchiectasis patients with *P. aeruginosa* isolation.

VOCs, such as 10-heptadecenoic acid, heptadecenoic acid, longifolene, and decanol were highly upregulated in the hypoxia group. However, there was no significant difference in levels of pentadecanoic acid, myristic acid, palmitoleic acid, and hexadecenoic acid between normoxia and hypoxia groups.

VOCs, such as pentadecanoic acid, 13-octadecenoic acid, octadecenoic acid, phenol, and myristic acid were highly upregulated in bronchiectasis patients with *P. aeruginosa* isolation. However, there was no significant difference in palmitoleic acid and hexadecenoic acid between *P. aeruginosa* (+) and *P. aeruginosa* (–) groups. Among those VOCs, myristic acid, pentadecanoic acid, hexadecanoic acid, and heptadecylic acid were acid compounds. A previous study indicated that myristic acid with anti-virulence properties may increase the pathogenicity of *P. aeruginosa* in a murine cutaneous infection model (31), which could explain the increase of myristic acid level in the *P. aeruginosa* (+) group of patients with bronchiectasis. Longifolene is a naturally occurring tricyclic sesquiterpene (32), and it has antimicrobial activities (33). Our study revealed that the longifolene level was the lowest in the mild group compared with that in the moderate and severe groups by BSI risk stratification, while the underlying mechanism needs further study. Kuo et al. found that octadecenoic acid could be produced from oleic acid conversion by strains of *P. aeruginosa* (34), which explained the high level of octadecenoic acid in the *P. aeruginosa* (+) group. A meta-analysis showed that elevated levels of pentadecanoic acid and heptadecanoic acid were associated with a lower risk of cardiovascular disease (35). However, as these two acids in our study were observed in EBC, rather than in serum, the effects may be different. In addition, pentadecanoic acid acted as a novel histone deacetylase 6 (HDAC6) inhibitor, and it promoted the acetylation of α-tubulin in MCF-7 breast and A549 lung cancer cells dose-dependently (36). Palmitoleic acid could form calcium palmitate or magnesium palmitate, thereby resulting in the formation of biofilms as bacterial cells use these salts as a carbon source for their growth (37). However, in the present study, palmitoleic acid showed no significant difference in any subgroups. Further studies should concentrate on the functions of those differentially expressed VOCs of bronchiectasis in the future.

The levels of 10-heptadecenoic acid and longifolene were significantly upregulated in the VOCs of the hypoxia group. Additionally, the levels of 13-octadecenoic acid and phenol were significantly upregulated in the VOCs of patients with *P. aeruginosa* isolation. In contrast, the levels of 10-heptadecenoic acid, heptadecanoic acid, octadecanoic acid, pentadecanoic acid, myristic acid, and longifolene were downregulated in the group with a lower E-FACED score, of which, the most significantly downregulated level in VOCs belonged to 10-heptadecenoic acid. The role of VOCs in the development of bronchiectasis has not yet been fully clarified. Mazzatenta et al. (38) suggested that VOCs could be a promising biomarker for hypoxemia, which was consistent with our findings. Bregy et al. suggested that the characteristic metabolites in COPD patients were different from those in healthy controls (39). According to a previous study, lipid synthesis was improved in the exacerbation of COPD (40). This phenomenon might be explained by the fact that the hypoxemia could affect the cell metabolism (41). Our study suggested that 10-heptadecenoic acid might be a valuable marker to predict hypoxemia in patients with bronchiectasis.

Furthermore, *P. aeruginosa* isolation could influence VOCs. van Oort et al. established a rat pneumonia model and found that *P. aeruginosa* infection could affect the profiles of the VOCs (42). We used OPLS-DA and logistic regression analysis to identify the biological roles of VOCs in bronchiectasis patients with hypoxia or with *P. aeruginosa* isolation. The OPLS-DA plot revealed samples from patients with hypoxia or normoxia, and those from *P. aeruginosa* (+) or *P. aeruginosa* (–) were separately distributed. This provided further evidence for the significant differences in VOCs between the hypoxia and normoxia groups, and between *P. aeruginosa* (+) and *P. aeruginosa* (–) groups. Moreover, the levels of 10-heptadecenoic acid, heptadecanoic acid, longifolene, palmitoleic acid, pentadecanoic acid, 13-octadecenoic acid, octadecenoic acid, hexadecanoic acid, myristic acid, phenol, and decanol were all different in the univariate analysis for the prognosis of bronchiectasis patients with hypoxia. The multivariate analysis was employed to assess factors influencing PaO_2_ level in patients with bronchiectasis, and it was revealed that among VOCs, 10-heptadecenoic acid might be the most important factor in the development of bronchiectasis. The multivariate analysis of *P. aeruginosa* isolation also suggested that octadecenoic acid and phenol might be the most important independent factors influencing the *P. aeruginosa* isolation. The upregulated level of octadecenoic acid in patients with *P. aeruginosa* isolation might be associated with its antibacterial activity (43).

The present study has some limitations. Firstly, the results of this study cannot be generalized because of the small sample size. Secondly, because there are individual differences among subjects, some of the statistical changes be observed are likely driven by outliers. Thirdly, no validation cohort was involved, indicating the necessity of involvement of a validation cohort to confirm the relationship between VOCs in EBC and the severity of bronchiectasis. Fourthly, no follow-up was conducted. Thus, it is essential to conduct additional large-scale multicenter studies with a long-term follow-up to eliminate the above-mentioned shortcomings. In addition, some of the VOCs aforementioned, such as 10-heptadecenoic acid, longifolene, decanol, 13-octadecenoic acid, pentadecanoic acid, and myristic acid are necessary for further research on the mechanism in the process of bronchiectasis infected by P. aeruginosa.

## 5 Conclusions

In summary, the present study showed the correlation between VOCs in EBC and the severity of bronchiectasis. The predictive capability of specific VOCs for hypoxia and *P. aeruginosa* isolation in bronchiectasis patients was revealed. In addition, 10-heptadecenoic acid and age were found as independent prognostic factors for bronchiectasis patients with hypoxia. Octadecenoic acid, phenol and gender were independent prognostic factors for bronchiectasis patients with *P. aeruginosa* isolation. Future studies are urgently needed to investigate the specific mechanisms.

## Data availability statement

The original contributions presented in the study are included in the article/Supplementary material, further inquiries can be directed to the corresponding author.

## Ethics statement

The studies involving humans were approved by the Ethics Committee of Shanghai Pulmonary Hospital (Approval No. K21-316). The studies were conducted in accordance with the local legislation and institutional requirements. The participants provided their written informed consent to participate in this study.

## Author contributions

S-YG: Writing – original draft, Conceptualization, Data curation, Funding acquisition, Investigation, Methodology, Writing – review & editing. H-WL: Writing – review & editing, Investigation, Project administration. J-WB: Writing – review & editing, Investigation, Methodology. J-WY: Writing – review & editing, Data curation, Investigation. BM: Writing – review & editing, Data curation, Investigation. LY: Writing – review & editing, Data curation, Investigation, Methodology. J-FX: Writing – review & editing, Conceptualization, Funding acquisition, Methodology, Resources, Validation, Visualization.
